# SARS-CoV-2-Induced Amyloidgenesis: Not One, but Three Hypotheses for Cerebral COVID-19 Outcomes

**DOI:** 10.3390/metabo12111099

**Published:** 2022-11-11

**Authors:** Carlos-Alberto Gonçalves, Larissa Daniele Bobermin, Patricia Sesterheim, Carlos Alexandre Netto

**Affiliations:** 1 Programa de Pós-Graduação em Ciências Biológicas: Bioquímica, Instituto de Ciências Básicas da Saúde, UFRGS, Porto Alegre 90035-003, Brazil; 2 Programa de Pós-Graduação em Neurociências, Instituto de Ciências Básicas da Saúde, UFRGS, Porto Alegre 90035-003, Brazil; 3 Centro Estadual de Vigilância Sanitária do Rio Grande do Sul (CEVS-RS), Porto Alegre 90450-190, Brazil

**Keywords:** amyloid, astrocyte, Aβ, COVID-19, fibrin, serum amyloid A, SARS-CoV-2

## Abstract

The main neuropathological feature of Alzheimer’s disease (AD) is extracellular amyloid deposition in senile plaques, resulting from an imbalance between the production and clearance of amyloid beta peptides. Amyloid deposition is also found around cerebral blood vessels, termed cerebral amyloid angiopathy (CAA), in 90% of AD cases. Although the relationship between these two amyloid disorders is obvious, this does not make CAA a characteristic of AD, as 40% of the non-demented population presents this derangement. AD is predominantly sporadic; therefore, many factors contribute to its genesis. Herein, the starting point for discussion is the COVID-19 pandemic that we are experiencing and how SARS-CoV-2 may be able to, both directly and indirectly, contribute to CAA, with consequences for the outcome and extent of the disease. We highlight the role of astrocytes and endothelial cells in the process of amyloidgenesis, as well as the role of other amyloidgenic proteins, such as fibrinogen and serum amyloid A protein, in addition to the neuronal amyloid precursor protein. We discuss three independent hypotheses that complement each other to explain the cerebrovascular amyloidgenesis that may underlie long-term COVID-19 and new cases of dementia.

## 1. Background

We have been observing that at least one-third to more than half of COVID-19 patients have neurological symptoms and/or signs, ranging from headaches to mood and anxiety disorders to strokes [1,2,3,4,5]. Many of these changes persist after remission of the infection, indicating that viral aggression or the response to aggression leaves residues. The clinical picture that is being characterized is called post-COVID-19 syndrome, or long COVID-19. These residues can involve cognitive deficits and even a greater susceptibility to cerebrovascular events (ischemic or hemorrhagic) [5,6,7].

Just as aggression and neurological manifestations are different between individuals, we can also conceive of different forms of persistence of central nervous system (CNS) injury. This phenomenon is complex and involves not only viral aggression, but also the susceptibility of individuals, age, preexisting comorbidities, and even medical intervention with anti-inflammatory drugs, oxygen therapy, and prolonged sedatives [1,8,9]. The literature on the subject is vast and growing. Our intention is to review and stitch together some ideas about the underlying mechanisms of the brain damage that is associated with SARS-CoV-2 infection [5,6].

### But What Is the Focus of This View?

From the beginning, it was recognized that the endothelium, due to its expression of angiotensin-converting enzyme 2 (ACE2), could explain the multisystemic aggression of SARS-CoV-2 and that endothelial dysfunction could be actively contributing to the severity of cases due to the production of cytokines, formation of clots, the worsening of hypoxemia, and tissue ischemia. In the CNS, the endothelium has a close structural and functional relationship with astrocytes (which also express ACE2), making these cellular elements essential for understanding primary and long-term brain changes in response to viral injury.

Herein, we will address three hypotheses of biochemical alterations that involve the triggering and/or worsening of the formation of amyloid-like protein aggregates (amyloidosis) by SARS-CoV-2 infection and could explain, in part, the more severe and/or lasting neuropsychiatric manifestations of COVID-19. The first of these and the most discussed in the literature is the alteration in the processing of amyloid precursor protein (APP), in terms of the neuronal production of amyloid peptides and the subsequent clearance by astrocytes, microglia, and endothelial cells. This event is linked to perivascular amyloid deposition, which facilitates the ischemic and hemorrhagic events observed. The second involves a peripheral phenomenon, external to the CNS, where amyloid clots form from fibrinogen and also eventually facilitate ischemic and hemorrhagic events in the CNS. Finally, we will discuss serum amyloid A (SAA), an acute-phase response (APR) protein of the liver that could also be involved in the formation of atypical clots and disruption of the blood–brain barrier (BBB) that is observed in patients, but could additionally contribute to vascular amyloidgenesis, as it is produced by astrocytes. In fact, all these hypotheses are available in the literature. Here, we emphasize these hypotheses and the role of astrocytes from the perspective of contributing to understanding the possible consequences of the SARS-CoV-2 pandemic on cerebral amyloidgenesis.

## 2. Amyloid Beta Peptide Deposition

The first hypothesis brings together a collection of information suggesting that SARS-CoV-2 infection alters the brain metabolism of APP. Neurons express (although they are not the exclusive producers of) APP, the function of which is unknown. However, the metabolism of APP leads to the formation of peptides, some of which are potentially neurotoxic, such as amyloid beta peptides 1–40 and 1–42 (Aβ). An imbalance between the production and clearance of these peptides can result in Alzheimer’s disease (AD). These peptides can aggregate into oligopeptides, change their helical conformation to beta-pleated sheets, and eventually deposit, forming an amyloid beta-fibrillar deposit [10,11]. 

It should be mentioned here that amyloid deposition can occur with many other proteins (such as immunoglobulins and apolipoproteins). These proteins lose their globular structure and acquire a fibrillar structure, where beta-pleated sheets are stacked, allowing recognition by dyes, such as Congo Red and Thioflavin T. The name amyloidosis, although inappropriate, was given to these extracellular deposits by Rudolf Virchow in the 19th century, as he believed them to be starch-like (amylo, from Greek) [12,13].

In the case of AD, Aβ deposition characteristically occurs in the brain parenchyma and is denominated as neural or senile plaques, which preferentially occur in the neocortex and hippocampus. It is not our intention to discuss, in detail, the formation or clearance of Aβ or amyloid deposition (see [14,15] for a review). This amyloid deposition also occurs around small brain vessels as CAA. In fact, perivascular amyloid deposition occurs in up to 90% of AD cases and up to 40% of older non-demented individuals [16]. CAA is a major cause of spontaneous intracerebral hemorrhage and is, in fact, commonly associated with cortical–subcortical microbleeds. The high presence of CAA in AD has led to a discussion as to whether perivascular amyloid deposition precedes or follows parenchymal deposits. Regardless of this issue, we will focus here on perivascular deposition. Although current thinking indicates that it is improbable that perivascular amyloid deposition is not biochemically linked to parenchymal deposition, it is important to mention that CAA is not exclusively Aβ deposition [17,18].

**Figure 1:**
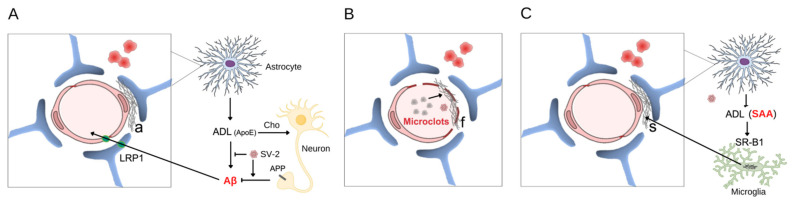
Vascular amyloid deposition induced by SARS-CoV-2. The intimate relationship between the endothelial cell (pink) and astrocyte feet (blue) that envelop the endothelial surface is indicated in the 3 panels. The stains (in red) next to the vessel, in all panels, represent microbleeds and emphasize that, in addition to the ischemic event caused by the amyloid deposit, a hemorrhagic event is associated with vascular fragility. In panel (**A**), a neuron is represented (yellow) with a membrane amyloid precursor protein (APP) and its amyloid peptide fragments (Aβ). The production of astrocyte-derived lipoprotein (ADL), containing ApoE, which is involved in cholesterol (Cho) transport and Aβ clearance, is shown, as well as the membrane transporter for Aβ (LRP1) in the astrocyte and endothelium. With virus-induced dysfunction, there is an accumulation of amyloid (a) between the astrocyte and the endothelium. In panel (**B**), the amyloid microclots of fibrin and the fibrinoid deposition (f) on the vascular surface are represented. In (**C**), the non-Aβ amyloid deposit of SAA (s) is represented, where SAA comes from the periphery and from the astrocyte (via ADL), which initially undergoes fibrillogenesis in macrophages (microglia in the CNS are in green). SV-2 = SARS-CoV-2.

Note that the brain endothelium is closely surrounded by the feet of astrocytes, forming the BBB [19]. Astrocytes play an active role in the clearance of the beta-amyloid produced by neurons, both by producing Aβ proteases (e.g., neprilysin) and by removing those in a process that involves apolipoprotein E (ApoE), which is also produced by astrocytes, and low-density lipoprotein receptor-related protein 1 (LRP1) membrane transporter. This supports the idea that astrocytic dysfunction precedes or accompanies amyloid deposition [20]. In fact, changes in glial fibrillary acidic protein (GFAP), the main astrocyte marker, are present long before cognitive signs of disease [21] and are directly related to cognitive impairment [22]; see Figure 1A. Amyloid deposition occurs between the endothelium and the astroglial layer in capillary vessels, but spreads throughout the vascular wall in small vessels.

**Figure 2:**
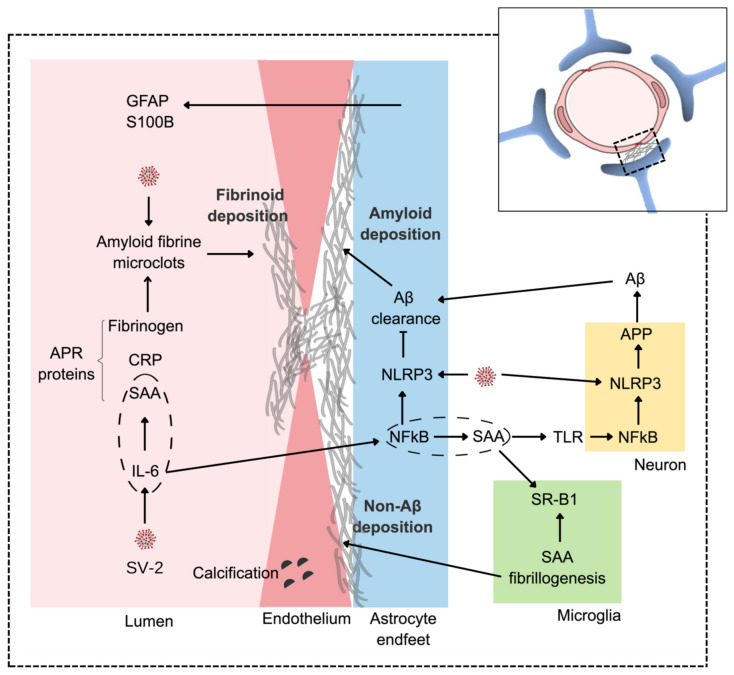
An integrative view of amyloid vascular deposition. SARS-CoV-2 (SV-2) triggers a peripheral hypercytokinemia led by IL-6, which, in the liver, leads to exacerbated production of APR proteins, including C-reactive protein, SAA, and fibrinogen. In the CNS, the virus directly affects endothelial cells and astrocytes compromising the BBB by inducing and facilitating vascular fibrinoid deposition. The astroglial response to SARS-CoV-2 and/or hypercytokinemia may be accompanied by increases in serum GFAP and S100B. The virus (via NLRP3) also increases APP metabolism, generating more Aβ. With astroglial dysfunction (also involving NLRP3), there is a reduction in Aβ clearance, leading to perivascular amyloid deposition. Note that IL-6, centrally, leads to an increase in astroglial SAA, which, in turn, could amplify APP neuronal processing through the activation of the TLR/NF-KB/NLRP3 pathway. Dashed ellipses emphasize the production of SAA by IL-6 in the hepatocytes (not shown) and astrocytes. Elevated peripheral production of SAA can accentuate fibrinoid deposition, while astroglial production can lead to non-AB amyloid deposition, which, in the CNS, involves the microglial scavenger receptor B1 (SR-B1).

Astrocytes, like endothelial cells, are directly affected by SARS-CoV-2 [19,23,24,25], which may compromise their ability to remove Aβ, leading to CAA. Astrocytic involvement in COVID-19 can be confirmed by elevated serum levels of specific markers, GFAP, and S100 calcium-binding B protein (S100B). BBB dysfunction could be linked to ischemic and hemorrhagic events in individuals infected with SARS-CoV-2 [26,27]. There are reports of an increase in ACE2 in AD patients [28,29], as well as a decrease in expression [30]. Virus entry, involving ACE2 anchoring and endocytosis, would result in a reduction in the Ang1-7-mediated anti-inflammatory response in endothelial cells and astrocytes [31,32]. Furthermore, in silico analysis showed that neuronal APP processing may be reduced, but that inflammatory upregulators of this processing may be increased [33]; see Figure 2. There are also data suggesting that SARS-CoV-2 activates NLR family pyrin domain-containing 3 (NLRP3) [34], which could increase APP processing [35,36] and negatively modulate Aβ clearance [37,38]. Therefore, based on this collection of information, we can say that SARS-CoV-2 may worsen the conditions of individuals affected by AD and/or CAA [39,40] without ceasing to think that the virus itself could trigger the process in many individuals. The epidemiological data that are beginning to be obtained confirm a greater impact of COVID-19 on patients with dementia (e.g., [41]), and we may soon see an increase in post-COVID-19 AD cases.

## 3. Amyloid Fibrin Clots

The second hypothesis is based on the formation of an atypical clot, followed by the deposition of an amyloid fibrin in the vessels [42]. A fibrin clot is a polymer of fibrinogen, a coil-coiled hexameric soluble protein (two alpha, two beta, and two gamma chains). The sequential removal of N-terminal fragments of the alpha and beta chains of fibrinogen by the action of thrombin allows its polymerization and entanglement, which can be reinforced by cross-links that are catalyzed by a transglutaminase (see [43,44] for a review). Commonly, the clot structure can be characterized by the diameter of the tangled fibers and the pores in the structure.

Some atypical clots, initially described as denser (without pores), were more recently characterized as “amyloid” fibrin clots induced by the lipopolysaccharide (LPS) produced by Gram-negative bacteria [45,46]. Both their insolubility and beta-pleated sheet structure (recognized by Congo red) allow them to be referred to as amyloid clots. In addition to LPS, other inducers can lead to the generation of atypical clots, such as lipoteichoic acid (derived from gram-positive bacteria) [47], estrogens [48], serum amyloid A protein (SAA) [49] and, more recently, the SARS-CoV-2 protein [50].

In fact, since the beginning of the COVID-19 pandemic, it has been noticed that the severity of cases was accompanied by an increase in blood coagulability, as indicated by the increase in fibrinogen, which is an APR protein, and by the presence of D-dimers, which are proteolytic products of fibrin, catalyzed by plasmin [51]. These clots are often referred to as atypical, as they resist treatment with some anticoagulants [52]. This coagulopathy is associated with the thrombotic and ischemic events of COVID-19 in the lungs, brain, and kidneys. However, vascular events are not purely ischemic, as viral invasion affects endothelial integrity, also leading to hemorrhagic events [7]. 

Based on these changes, it has been proposed that amyloid fibrin clots may underlie the systemic and long-term effects of COVID-19 (see [42] for a detailed and well-supported explanation of this hypothesis). See Figure 1B, where amyloid microclots associated with neurovascular damage may be producing the ischemic events and cerebral microbleeds that are observed in COVID-19. In fact, these amyloid microclots may underlie longer-lasting changes in COVID-19, challenging our methods of diagnosis and treatment with anticoagulants. The authors point out that other viruses that cause Dengue and Zika, and that also cause CNS disorders, are capable of interfering with the clotting mechanism and causing these atypical clots. This may contribute to explaining the neuropsychiatric symptoms of those diseases.

## 4. Serum Amyloid A Protein

As previously mentioned, SAA can induce the formation of atypical clots [49], and the hypothesis that this protein, being an APR protein, could be a mediating part of the toxicity of SARS-CoV-2 was raised [53]. In fact, this protein could be a good marker of the severity of COVID-19. In addition, it could contribute acutely and actively to the pathogenesis of the disease, as SAA can, in addition to contributing to atypical clots, cause the agglutination of red blood cells, aggravating hypoxemia [49]. SAA can also disturb the transport of cholesterol, aggravating cardiovascular atheromatous changes [54]. 

There are four isoforms of the SAA protein, 1, 2 3, and 4 (see [55] for a review). Isoforms 1 and 2 are more abundant and probably organized as hexamers, wherein each subunit has an approximate molecular weight of 11 kDa. SAA is quite insoluble and circulates associated with high-density lipoprotein (HDL), and is, therefore, referred to as an apolipoprotein. The SAA 4 isoform is constitutive (i.e., it does not respond as an APR protein). Several receptors for SAA have been identified, including scavenger-receptor SR-B1 (involved in cholesterol efflux and removal of SAA), toll-like receptors 2 (TLR2) and 4 (TLR4), and receptor for advanced glycated end-products (RAGE) (all enhancing immune activation). Intracellular accumulation can lead to fibrillogenesis in a pH-dependent manner, causing systemic AA amyloidosis [13].

In addition to hepatocytes, this protein is produced in other cells, such as adipocytes and macrophages. In the CNS, SAA 1 and 2 are found in microglia, oligodendrocytes, and astrocytes, where they respond to alarmins, cytokines, and LPS [56,57]. An increase in astroglial SAA 3 has been described in APP transgenic mice [58]. Furthermore, elevated levels of SAA were detected in the cerebrospinal fluid (CSF) of AD patients [59] and in the brain tissue of AD patients [58,60,61]. Elevated astroglial SAA could affect BBB [62], confirming the possibility that elevated peripheral SAA causes the rupture of this barrier [61].

In COVID-19, correlations show greater disease severity in patients with high serum SAA levels [63,64,65,66]. Accordingly, patients with high serum levels of SAA (diabetics and obese pre-diabetics) were more prone to hospital admission and mortality when infected with SARS-CoV-2 [67]. There is no reason to suspect that neural cells are not, like peripheral cells, responding to SARS-CoV-2 aggression with more SAA production. 

Interestingly, Jana and colleagues, reported that the C-terminal segment of the E (from envelope) protein of SARS-CoV-2 was capable of inducing SAA fibrillogenesis [68], which could underlie post-COVID systemic AA amyloidosis [69]. Furthermore, here, we postulate that astroglial SAA may be directly involved in brain amyloid deposition. This could even be a fourth hypothesis. Indeed, current data support the possibility that glial SAA contributes to a non-Aβ CAA and the long-lasting neurological changes from COVID-19; see Figure 1C. Astrocytes, stimulated by IL-6, produce SAA, which, in turn, in the presence of SARS-CoV-2, could produce amyloid deposits [50]. Indeed, increases in SAA have been observed in brain tissue and the CSF of AD patients. However, as SAA causes damage to the BBB, it is difficult to determine the origin (peripheral or cerebral) of this CSF SAA. However, despite the increase in serum SAA, so far, no increase in SAA in the CSF of patients with COVID-19 or post-COVID-9 has been described.

For this hypothesis to work in the CNS, it is also necessary to consider that SAA probably circulates with the astrocyte-derived lipoprotein that is produced by astrocytes and that is normally captured by neurons through specific receptors [70]. Peripherally, SAA is probably taken up by macrophages via SR-B1 receptors, where primordial fibrillogenesis probably occurs in lysosomal vesicles [55], initiating systemic AA amyloidosis. In the CNS, the removal of SAA and primordial fibrillogenesis would involve the microglia.

## 5. Complementarity between Hypotheses and with Other Biochemical COVID-19 Changes

The three hypotheses presented are not antagonistic and, in fact, can complement each other. Together, they expand our understanding of the neurological changes in COVID-19 and may explain, in part, the manifestations of long-term COVID-19. The first hypothesis is supported by the increased neuronal processing of APP and reduced clearance of Aβ peptides due to the dysfunction induced directly by SARS-CoV-2 in astroglial and endothelial cells. The Aβ deposit compromises communication between these cells, resulting in damage to the neurovascular unit. Endothelial damage favors clot formation, which is the basis of the second hypothesis. Microclots, in turn, reinforce endothelial damage, allowing for ischemic and hemorrhagic events. These clots are atypical due to the direct action of SARS-CoV-2, but may also be atypical due to the increased presence of SAA from the liver and macrophages, which is the basis of the third hypothesis. Reinforcing this hypothesis, we postulate that the neural increment of SAA induced by SARS-CoV-2 leads to a cerebral amyloid deposit. Note that SARS-CoV-2 induces an increase in SAA and also favors its fibrillogenesis—peripheral (atypical clots) and central (non-Aβ amyloid deposition); see integrative Figure 2.

Other observed biochemical changes may contribute to the fibrillogenesis postulated in these hypotheses. We can highlight two alterations that are also associated with the severity of the disease—metabolic acidosis [71] and hypocalcemia [72]. In COVID-19, many factors converge to lead to metabolic acidosis, such as hypoxemia and renal impairment. In general, fibrillogenesis is favored by acidosis and, additionally, in the case of Aβ peptides, there is an apparent impairment of astroglial clearance by acidosis [73]. Moreover, in the case of SAA, the expression of the microglial scavenger receptor SR-B1 increases in acidic media [73], in turn increasing microglial SAA uptake and initiating its fibrillogenesis, which is initially intracellular [74].

Another aspect that may be involved is the hypocalcemia that occurs in the most severe cases of the disease. Many factors may be involved in this event, such as carboxylic acid sequestration (of lactate, ketogenic bodies, and free fatty acids), vitamin D decrease due to kidney damage, and parathyroid hormone changes caused by virus or drug interaction, but none of these are sufficiently convincing [72,75]. However, we emphasize that the amyloidgenic hypotheses raised herein may explain neuroCOVID-19. Low calcium may reflect vascular deposition, in which endothelium involvement is key. Vascular deposition is an important element in chronic kidney disease, but during COVID-19, coronary artery vascular calcification can also be seen, literally, in the most severe cases (e.g., [76,77]). The inflammatory process and ischemia cause the endothelium to slough off and contribute to thrombus formation and bleeding events [16], but it also causes endothelial–mesenchymal conversion [78], leading to the calcification of the endothelium itself and inducing mesenchymal changes and calcification in the vascular media layer. Therefore, hypocalcemia reflects, in part, endothelial dysfunction/vascular calcification, which contributes to the formation of clots and atypical microclots, cerebral ischemia, and reduced cerebral Aβ and SAA clearance.

## 6. Concluding Remarks

1. SARS-CoV-2 causes amyloidgenesis, and this may underlie the lasting effects of post-COVID-19;
2. In the CNS, the effect is induced directly by viral proteins on fibrin and SAA fibrillogenesis, or indirectly on Aβ peptides, altering the production/clearance balance;
3. The hypotheses discussed here of amyloidgenesis are complementary and converge, leading to ischemic and/or hemorrhagic events, which compromise the functionality of the CNS;
4. These hypotheses reinforce the importance of astrocytes and the brain endothelium for brain integrity;
5. Here, we emphasize perivascular amyloidosis induced by SARS-CoV-2, but it should be remembered that amyloidosis—as an unfolded protein disorder—is a ubiquitous phenomenon that underlies many diseases. Thus, it may be postulated that many other manifestations of COVID-19 may involve disorders of unfolded proteins;
6. Infection by SARS-CoV-2, like that by some other pathogens, is capable of aggravating existing imbalances (co-morbidities) and/or even causing disorders in protein homeostasis. Thus, many non-communicable diseases (such as AD, diabetes mellitus, and stroke) can be exacerbated and even triggered by communicable disease agents;
7. In the 19th century, diseases caused by infectious agents, due to vaccines and antibiotics, lost epidemiological relevance to non-communicable diseases. Apparently, the epidemiology of communicable diseases may recuperate its social and economic importance, shedding light on the dialectical essays on health by Richard Lewontin and Richard Levins in the book *Biology Under Influence*.

## Data Availability

The data presented in this study are available in the main article.
